# Area postrema syndrome with linear enhancement along the surface of the brainstem and fourth ventricle in autoimmune GFAP astrocytopathy

**DOI:** 10.1186/s12883-023-03126-5

**Published:** 2023-02-20

**Authors:** Xinming Liang, Yaoyao Shen

**Affiliations:** 1 Department of Neurology, Nanyang Central Hospital, Nanyang, Henan Province China; 2 Department of Neurology, Jiangxi Provincial People’s Hospital, The First Affiliated Hospital of Nanchang Medical College, No. 92 Aiguo Road, Donghu District, Nanchang, 330006 Jiangxi Province China

**Keywords:** Glial fibrillary acidic protein, Area postrema syndrome, Magnetic resonance imaging

## Abstract

**Background:**

Glial fibrillary acidic protein (GFAP) astrocytopathy, a novel autoimmune disease of the nervous system, was first defined in 2016. To our knowledge, area postrema syndrome (APS) with linear enhancement along the surface of the brainstem and fourth ventricle is extremely rare in this disorder.

**Case presentation:**

A Chinese woman presented with intractable nausea and vomiting after onset of flu-like symptoms. Brain magnetic resonance imaging (MRI) disclosed abnormal signal intensities in the dorsal medulla oblongata including area postrema. Besides, linear enhancement surrounding the surface of the brainstem and fourth ventricle was visualized after gadolinium injection. Cerebrospinal fluid (CSF) analysis showed increased cell count and protein. A cell-based assay was positive for anti-GFAP IgG in CSF. She was diagnosed with autoimmune GFAP astrocytopathy and treated with high-dose glucocorticoid. The patient received a quick recovery with entire resolution of the initial abnormalities.

**Conclusions:**

Isolated APS can be the initial manifestation of autoimmune GFAP astrocytopathy. Linear enhancement surrounding the surface of the brainstem and fourth ventricle is another neuroradiological hallmark.

## Background

Glial fibrillary acidic protein (GFAP), the predominant intermediate filament protein in mature astrocytes, plays important roles in supporting the neurons, providing integrity to the blood brain barrier (BBB), promoting synaptic plasticity, protecting neurons against neurotransmitter excesses, and coordinating neuronal activity [1]. In 2016, Lennon et al. [2] first defined autoimmune GFAP astrocytopathy, in which GFAP-IgG in serum and/or cerebrospinal fluid (CSF) detected by tissue-based assay and cell-based assay (CBA) was considered to be a specific biomarker. This disorder always manifests as a disabling corticosteroid-responsive meningoencephalomyelitis, involving optic nerve, subcortical white matter, ventricle, basal ganglia, thalamus, corpus callosum, brainstem, cerebellum, spinal cord, and meninges [3–5]. Area postrema syndrome (APS), which refers to episodes of intractable nausea, vomiting or hiccups mimicking gastrointestinal disorders, is one of the six core clinical characteristics of aquaporin-4 (AQP4)-IgG seropositive neuromyelitis optica spectrum disorder (NMOSD). However, APS as an initial manifestation of autoimmune GFAP astrocytopathy is rarely reported in the literature [6]. To our knowledge, linear perivascular radial gadolinium enhancement oriented to the ventricles on magnetic resonance imaging (MRI) is regarded as a hallmark of autoimmune GFAP astrocytopathy [2]. Whereas linear enhancement surrounding the surface of the brainstem and fourth ventricle is seldom described. Here, we report an interesting case who developed APS with positive GFAP antibodies in CSF and distinctive leptomeningeal enhancement along the surface of the brainstem and the fourth ventricle on postgadolinium T1-weighted image (T1WI).

## Case presentation

A previously healthy 30-year-old Chinese woman was admitted to local hospital owing to fever (a maximum of 38.7℃), headache, fatigue, and loss of appetite for one week. Initial laboratory tests yielded leukocytosis (12, 000 cell/μL), hyponatremia (128 mmol/L), hypokalemia (3.2 mmol/L), and a slight increase of erythrocyte sedimentation rate (30 mm/h) and C-reactive protein (12 mg/L). Chest computed tomography (CT) and abdominal ultrasonography revealed normal findings. She was initially administrated with intravenous acyclovir and cefotaxime due to consideration of infectious causes, as well as electrolyte supplements. Her body temperature gradually dropped below 37.2℃ within 5 days, but she complained of intractable nausea and vomiting on day 13 after disease onset. Although she received proton pump inhibitors and antiemetic drugs for the next 4 days, her vomiting worsened (10–15 episodes per day) and was concomitant with dizziness and diplopia. Therefore, the patient was transferred to our center in order to further diagnosis and treatment. On admission, her vital signs were a temperature of 36.7℃, a heart rate of 102 beats per minute, and blood pressure of 100/76 mmHg. Neurological examination demonstrated bilateral horizontal gaze-evoked nystagmus and positive Kernig’s sign, without focal neurological deficit or change in bladder and bowel habits. Deep tendon reflexes were present and symmetrical. Routine laboratory investigations including complete blood picture, clotting profile, C-reactive protein, erythrocyte sedimentation rate, thyroid hormones, tumor markers, and biochemistry revealed insignificant findings.

**Fig. 1:**
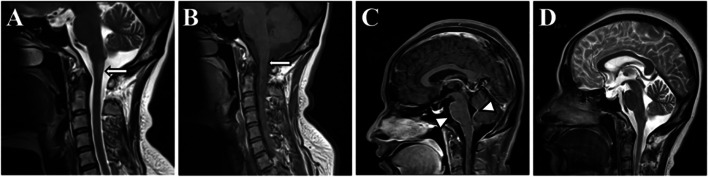
Sagittal MRI shows abnormal signal intensities in the dorsal medulla oblongata including area postrema (arrows), with hyperintense on T2-weighted image and hypointense on T1WI (**A**, **B**). Sagittal T1-weighted postgadolinium imaging demonstrates linear enhancement along the surface of the brainstem and fourth ventricle (**C**, arrowheads). Sagittal T2-weighted imaging reveals resolution of T2-hyperintense lesion (**D**)

On day 2 after admission, an upper digestive endoscopy was subsequently performed and there was no evidence of potential digestive system diseases, such as gastroesophageal reflux disease, gastroparesis or tumor. A brain and cervical spine MRI disclosed abnormal signal intensities in the dorsal medulla oblongata including area postrema, which were hyperintense on T2-weighted image and hypointense on T1WI (Fig. 1A, B). It was worth noting that distinctive linear enhancement surrounding the surface of the brainstem and fourth ventricle was visualized after gadolinium injection (Fig. 1C). Supratentorial or cerebellar periventricular linear radial enhancement was absent. Lumbar puncture revealed normal opening pressure. CSF analysis showed increased cell count (342 cells/mm3, 71% of lymphocytes) and protein (1.59 g/L), with normal glucose, chloride and oligoclonal bands. Microbiological investigations, including bacterial culture, acid-fast bacilli smear, T-spot, virus polymerase chain reaction, India Ink preparation, anti-HIV antibody, anti-syphilis antibody, were all unremarkable. Whole-abdomen CT did not detect any underlying malignant tumors. Work-up for autoimmune encephalitis-related panel, including anti-LGI1, anti-NMDAR, anti-CASPR2, anti-AMPAR, anti-GABA(B)R, anti-Hu, anti-Yo, anti-Ri, anti-amphiphysin, anti-CV2 and anti-Ma2 antibodies, were all negative both in serum and CSF. Antinuclear antibody spectrum screening of serum, including anti-nuclear antibodies (ANA), anti-extractable nuclear antigen (ENA), anti-DNA antibodies, anti-phospholipid antibodies, and antineutrophil cytoplasmic antibodies (ANCA), was unremarkable.

Based on the clinical presentation of intractable vomiting with MRI finding of area postrema involvement, the diagnosis of APS could be established. As there was no evidence of infectious encephalomyelitis or malignant condition, inflammatory demyelinating diseases of central nervous system (CNS) needed to be taken into account preferentially. CBA for GFAP antibodies were detected in both serum and CSF. Anti-GFAP antibody was only positive for CSF (1:32), while anti-AQP4 and anti-MOG antibodies were negative in both blood and CSF. Hence, the patient was finally diagnosed with autoimmune GFAP astrocytopathy and treated with high-dose glucocorticoid (methylprednisolone 1 g/day for 3 days) followed by oral prednisolone with gradually tapered off. The patient received a quick recovery and was discharged home without any residual symptoms. On day 15 after admission, a repeated MRI revealed entire resolution of the initial abnormalities (Fig. 1D). At last follow-up, 1 year later, no relapse had occurred.

## Discussion and conclusions

GFAP, one of the key components of the cytoskeleton of astrocytes, belongs to the family of intermediate filaments. It was initially detected in brains of patients with multiple sclerosis (MS) in 1971 [7]. In 1989, Reeves et al. first cloned the human GFAP gene, which was mapped to chromosome 17q21 and consisted of nine exons, eight introns, four alternative exons and two alternative introns [8]. GFAP and other intermediate filaments share a common structure characterized by three major domains: N-terminal head, central helical rod, and C-terminal tail.

Up to now, more than eight isoforms of GFAP protein have been identified and GFAPα is the most predominant [9]. Apart from supporting the neurons, GFAP has a much broader function in fundamental cellular processes, blood–brain barrier, myelination, synaptic plasticity, neurite outgrowth, injury/protection [1]. GFAP not only distributes in astrocytes, but also has a wide expression in non-glial and even non-CNS cells including chondrocytes, fibroblasts, myoepithelial cells, and liver stellate cells [1]. Using tissue-based immunofluorescence assay, GFAP-IgG staining depicts filamentous pattern on mouse brain, and predominates in pia, subventricular, periventricular, cortical surface, and perivascular regions [2].

Autoimmune GFAP astrocytopathy, a recently proposed entity, is defined as a corticosteroid-responsive meningo-encephalomyelitis with positive GFAP-IgG in CSF. Nowadays, the pathogenesis of GFAP astrocytopathy is poorly understood. It is difficult for GFAP-IgG to produce directly pathogenic effect because its target is an intracellular protein. Previously, animal model studies suggested that GFAP-specific CD8 T cells could avoid tolerance mechanisms before entering into the CNS and induce autoimmune damage [10]. Similar to autoimmune encephalitis, GFAP astrocytopathy is related to neoplasm. In a case series of 102 patients, neoplasia was found prospectively in 22%, 68% of which were ovarian teratoma [3]. In another study performed by Lennon et al., 6 of 16 patients had a neoplasm detected within 3 years of neurologic onset [1]. Immunohistochemical staining from the teratoma showed cytoplasm of the glial process in neuronal tissue and epithelial cells reacted strongly with GFAP-IgG [11]. Ectopic expression of GFAP by nervous tissue contained in the neoplasm contributes to breaking immune tolerance and triggering paraneoplastic neurological autoimmunity. In addition, some patients with GFAP antibody are associated with prodromal infection prior to onset of CNS symptoms, especially viral infection [3, 12]. In our case, despite negative result of a comprehensive microbiological investigation, the prodromal symptoms of fever and headache still unable to exclude potential viral infection. Unfortunately, this patient did not perform a next-generation sequencing (NGS) examination. The relationship between GFAP and prodromal infection remain unclear. There is limited information about the histopathological research of GFAP astrocytopathy. An autopsy case demonstrated diffuse inflammation involving the brain parenchyma, perivascular spaces, and leptomeninges, with prominent T-cells, macrophages, and activated microglia [13]. Histopathology analysis of a leptomeningeal biopsy specimen illustrated a mononuclear infiltration with macrophages and CD8 + T cells [11].

The predominant clinical syndrome of GFAP astrocytopathy is meningoencephalitis, followed by meningoencephalomyelitis, meningitis, myelitis, and optic neuropathy [3, 14]. Other clinical presentations include consciousness disturbance, dementia, seizures, autonomic nervous dysfunction, dyskinesia, and psychiatric symptoms [14]. In a French cohort study of patients with GFAP autoantibodies, abnormal hyperintensity lesions on brain T2-weighted and fluid-attenuated inversion recovery (FLAIR) images were widely distributed, involving in periventricular regions, brainstem, thalamus, internal and external capsule, basal ganglia, corona radiata and semiovale centrum, temporopolar area, limbic structures, hypothalamus, and corpus callosum. Area postrema involvement occurred in only one patient [14]. The APS, defined as otherwise unexplained and intractable episodes of hiccups, nausea, and vomiting, frequently occurs in NMOSD, but rarely in myelin oligodendrocyte glycoprotein-IgG-associated disorders (MOGAD) [15]. Analysis of an international database for AQP4-IgG-seropositive NMOSD showed the incidence of APS was found to be 9.4%—14.5% [16]. However, APS is rarely reported in MOGAD. In a cohort study included 173 patients with MOGAD, only 1 (0.6%) presented with APS [15]. In a case series of 50 adult patients with MOGAD, only one experienced APS [17]. Previously, APS was increasing reported as a noticeable manifestation of GFAP astrocytopathy. Whereas, the incidence of APS has not been well understood. To date, a total of 16 GFAP-IgG-positive patients presented with APS have been described in the literature [18]. Our case emphasizes that isolated APS can be the initial manifestation of autoimmune GFAP astrocytopathy. The area postrema involvement can be explained by GFAP-IgG preferentially targeting to ependymal and peri-ependymal regions in immunohistochemical staining. As far as we known, linear perivascular radial gadolinium enhancement oriented to the ventricles is a neuroimaging hallmark of GFAP astrocytopathy. In our case, however, this typical neuroimaging feature is absent and replaced by linear enhancement along the surface of the brainstem and fourth ventricle. Hence, our case highlights that this novel neuroradiological finding may be another marker for the disease diagnosis and should promote early consideration of GFAP-IgG testing. Pia enhancement is supported by the evidence of inflammation around small blood vessels in histopathology research, which leads to gadolinium leaking from the damaged BBB. Just as this case reported here, the enhancement disappeared entirely may benefit from rapid repairment of the BBB following glucocorticoid treatment.

In conclusion, a wide spectrum of neurological manifestations has been described in autoimmune GFAP astrocytopathy. This disorder should be considered in the differential diagnosis for patients who present with APS. If linear enhancement along the surface of the brainstem and fourth ventricle is found on brain MRI, GFAP-IgG should be timely detected to avoid misdiagnosis and delayed treatment.

## Data Availability

Not applicable.

## Abbreviations

GFAP: Glial fibrillary acidic protein
BBB: Blood brain barrier
CSF: Cerebrospinal fluid
CBA: Cell-based assay
APS: Area postrema syndrome
AQP4: Aquaporin-4
NMOSD: Neuromyelitis optica spectrum disorder
MRI: Magnetic resonance imaging
T1WI: T1-weighted image
CT: Computed tomography
CNS: Central nervous system
MS: Multiple sclerosis
NGS: Next-generation sequencing
FLAIR: Fluid-attenuated inversion recovery
MOGAD: Myelin oligodendrocyte glycoprotein-IgG-associated disorders
